# Review of the genus *Locastra* Walker, 1859 from China, with descriptions of four new species (Lepidoptera, Pyralidae, Epipaschiinae)

**DOI:** 10.3897/zookeys.724.13262

**Published:** 2017-12-21

**Authors:** Hua Rong, Houhun Li

**Affiliations:** 1 College of Life Sciences, Nankai University, Tianjin 300071, P. R. China

**Keywords:** Description, diagnosis, key, morphology, taxonomy

## Abstract

The genus *Locastra* Walker, 1859 from China is reviewed. Of the five species treated, four species are described as new: *L. nigrilineata* **sp. n.**, *L. solivaga* **sp. n.**, *L. subtrapezia* **sp. n.** and *L. viridis* **sp. n.** A diagnosis of *L. muscosalis* (Walker, 1866) is given. Photographs of adults and the genitalia are provided, along with a key to the five Chinese species treated.

## Introduction

*Locastra* Walker, 1859 was established in the Noctuoidea to accommodate four species: *L. maimonalis* Walker, 1859, *L. phereciusalis* Walker, 1858, *L. sagarisalis* Walker, 1858, and *L. haraldusalis* Walker, 1859. Only the first species, *L. maimonalis*, still remains in this genus. Hampson (1896a) designated *L. maimonalis* as the type species and transferred *Locastra* to the Epipaschiinae in the Pyralidae. He also placed *Eurois crassipennis* Walker, 1857, a nominal species not originally included in *Locastra*, as a senior synonym of *L. maimonalis* (see Fletcher and Nye 1984). Hampson (1896a) transferred *Taurica muscosalis* Walker, 1866 from Northern China to *Locastra*, and considered *Taurica sikkima* Moore, 1888 from India and *Locastra cristalis* Hampson, 1893 from Sri Lanka as its junior synonyms. Subsequently, other species were added to this genus: *L. pachylepidalis* from Bhutan (Hampson 1896b), *L. ardua* from Fiji (Swinhoe 1902), and *L. bryalis* from Vietnam (Joannis 1930).

*Locastra* currently comprises five species worldwide (Solis 1992; Nuss et al. 2003–2017) occurring in the Oriental Region, except *L. muscosalis* from China occurring in both Palaearctic and Oriental regions. Prior to this study, *L. muscosalis* was the only species recorded in China. The aim of the present paper is to review *Locastra* in China, including descriptions of four new species.

## Material and methods

Specimens examined in the present study were collected by light traps. Adults were examined using an Olympus SZX9 stereomicroscope. Permanent mounting methods of genitalia and venation follow the techniques introduced by Li (2002). Images of adults were taken with a Leica M205A stereomicroscope coupled with Leica Application Suite 4.2 software, and images of genitalia were prepared with a Leica DM750 microscope equipped with the same software and refined in Photoshop CS5.

All specimens studied, including the types of the new species, are deposited in the Insect Collection of Nankai University (NKU), Tianjin, China and the Biology Museum, Sun Yat-sen University, Guangzhou, Guangdong, China (SYSBM) as mentioned.

## Taxonomy

### Locastra

Taxon classificationAnimaliaLepidopteraPyralidae

Walker, 1859

Locastra Walker, 1859. Type species: Locastra maimonalis Walker, 1859.
Taurica Walker, 1866. Type species: Taurica muscosalis Walker, 1866.

#### Diagnosis.

*Locastra* can be distinguished externally by the large body and sub-globose scape extension in males; in male genitalia by the basally separated juxta; and in female genitalia by the usually distally S-shaped ductus bursae with longitudinal sclerotized ridges.

#### Generic characters.

**Figures 1–3.**
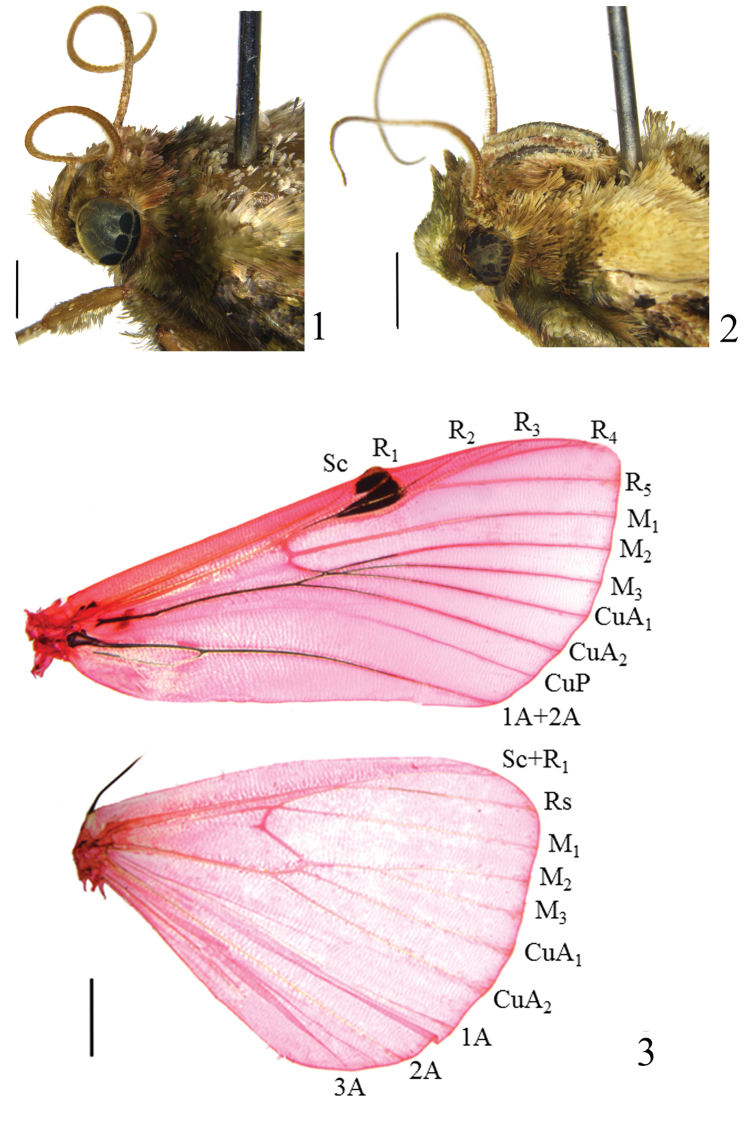
Heads and venation of *Locastra* spp. **1–2** Heads **1** *L. viridis* sp. n. **2** *L. nigrilineata* sp. n. **3** Venation of *L. muscosalis*, male, slide No. RH15249. Scale bars: 2.0 mm.

**Figures 4–8.**
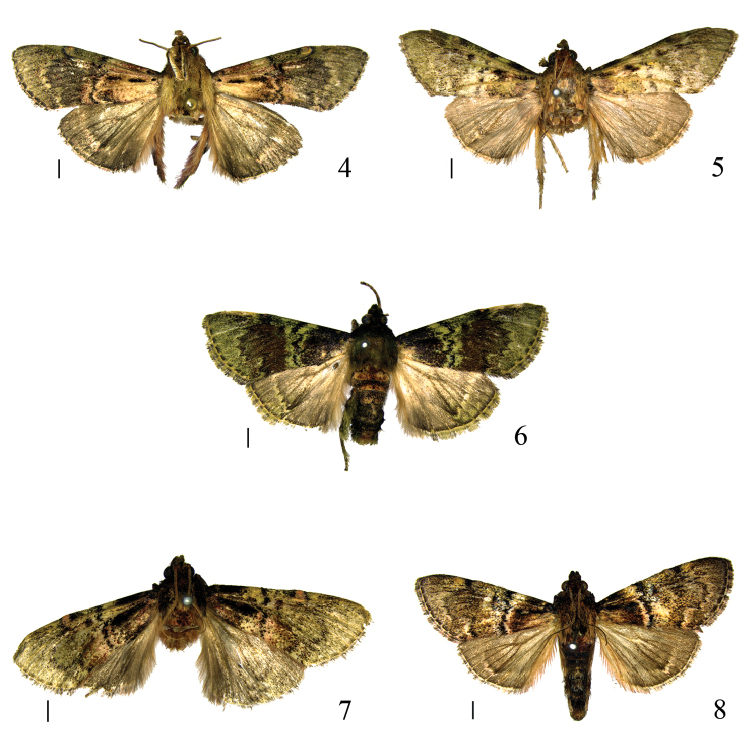
Adults of *Locastra* spp. **4** *L. nigrilineata* sp. n. holotype, male **5** *L. subtrapezia* sp. n. paratype, male **6** *L. viridis* sp. n. holotype, male **7** *L. solivaga* sp. n. paratype, male **8** *L. muscosalis*, male. Scale bars: 2.0 mm.

Body large, ranging from 29.0 to 50.0 mm. Head (Figs 1–2): Labial palpus upturned in male, slightly porrect in female, second segment with hair-like scales on inner side. Maxillary palpus short, compressed in both male and female. Antenna with cilia on ventral surface in both male and female; male usually with scape extension extremely short, sub-globose, developed in a few species. Forewing (Figs 4–8) usually with distinct antemedian and postmedian lines, with glandular swelling at costal margin before postmedian line; discal spot absent; discocellular spot small, represented by black tuft; tuft usually placed below middle of lower margin of cell. Wing venation (Fig. 3): Forewing with R_3_, R_4_ and R_5_ stalked, M_1_ from below upper angle of cell, M_2_, M_3_ and CuA_1_ from lower angle of cell, glandular swelling on costa causing veins around it to curve somewhat; hindwing with Sc+R_1_ and Rs adjacent, Rs and M_1_ from upper angle of cell, M_2_ and M_3_ from lower angle of cell, CuA_1_ from near lower angle of cell. Tibiae with long hair-like scales on outer side.

**Figures 9–13.**
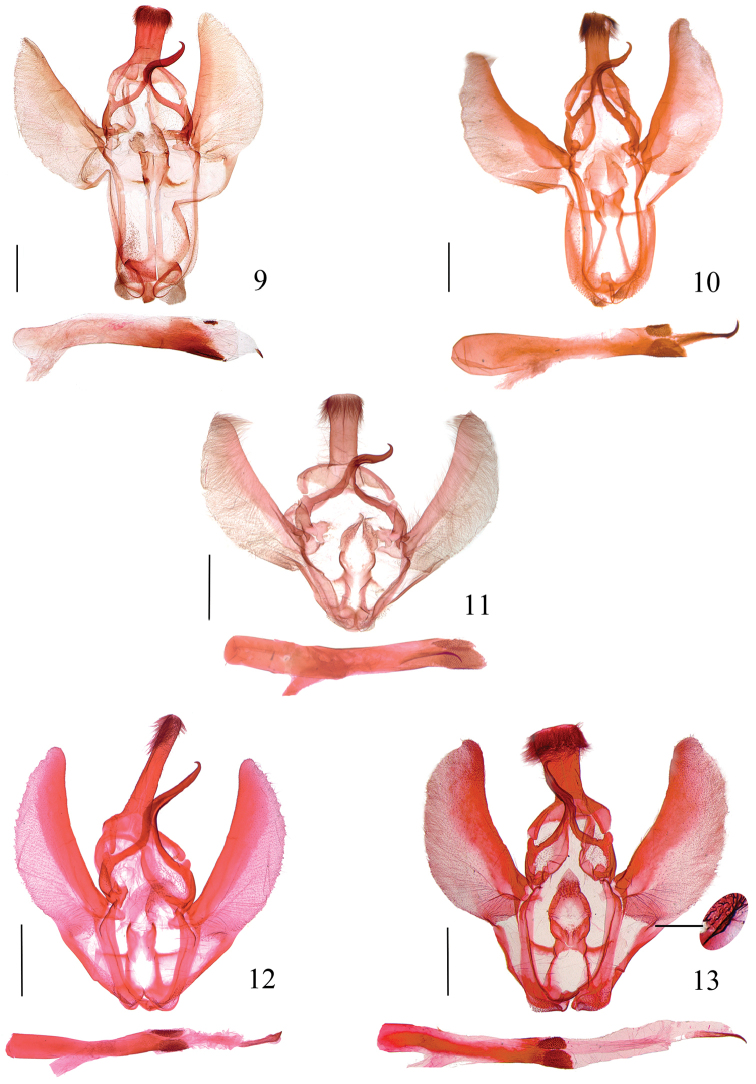
Male genitalia of *Locastra* spp. **9** *L. nigrilineata* sp. n. paratype, slide No. RH15425 **10** *L. subtrapezia* sp. n. paratype, slide No. RH16457 **11** *L. viridis* sp. n. paratype, slide No. RH15321 **12** *L. solivaga* sp. n. paratype, slide No. RH16484 **13** *L. muscosalis*, slide No. RH15249. Scale bars: 1.0 mm.

Male genitalia (Figs 9–13). Uncus sub-rectangular or trapezoidal. Gnathos distal process hooked. Valva with costa developed, ventral margin bluntly arched, with curved band from ventrobasal corner to base of juxta; costa and sacculus developed. Vinculum rather long in some species. Juxta separated basally, forming two well-sclerotized and narrowly banded lateral arms, merged medially or distally, usually concave on posterior margin; posterolateral lobes joined at apex; slender arms usually extending from lateral margin to vinculum in a right angle. Phallus with ovate sclerotized plates on ventral and dorsal surfaces before apex, with dense denticles; cornutus present or absent.

**Figures 14–16.**
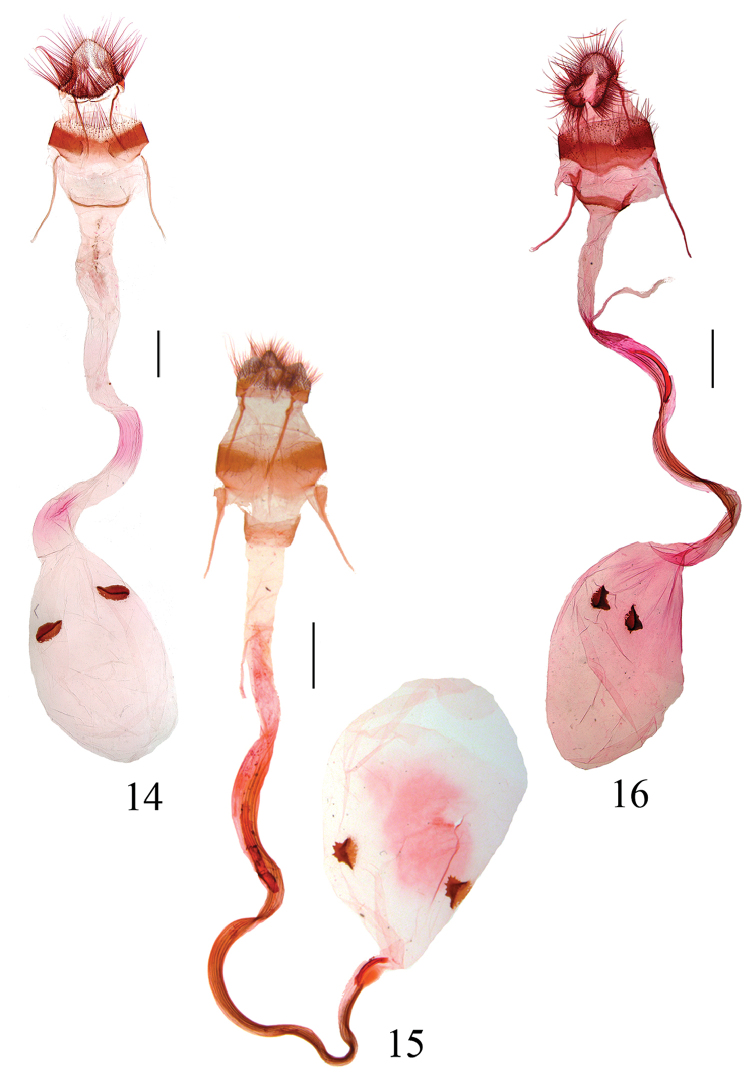
Female genitalia of *Locastra* spp. **14** *L. nigrilineata* sp. n. paratype, slide No. RH16152 **15** *L. viridis* sp. n., paratype, slide No. RH16460 **16** *L. muscosalis*, slide No. RH15424. Scale bars: 1.0 mm.

Female genitalia (Figs 14–16). Eighth tergite with anterior margin shallowly concave medially; eighth sternite narrow, membranous medially. Apophyses anteriores longer than apophyses posteriores, usually expanded basally. Antrum weakly sclerotized. Ductus bursae usually S shaped distally, with longitudinal sclerotized ridges. Corpus bursae ovate; signa two, ovate, with strongly sclerotized ridge medially.

**Key to *Locastra* species based on external characters and male genitalia**

**Table:** 

1	Vinculum approx 2.0 times length of uncus	**2**
–	Vinculum approx equal to length of uncus	**3**
2	Scape extension reaching mesothorax (Fig. 2)	***L. nigrilineata* sp. n.**
–	Scape extension extremely short, reaching before patagium (Fig. 1)	***L. subtrapezia* sp. n.**
3	Valva with triangular process at basal 1/3 of ventral margin (Fig. 13)	***L. muscosalis* (Walker)**
–	Valva without process at basal 1/3 of ventral margin	**4**
4	Uncus approx 5.0 times as long as maximum width (Fig. 12)	***L. solivaga* sp. n.**
–	Uncus approx 1.7 times as long as maximum width (Fig. 11)	***L. viridis* sp. n.**

### Locastra nigrilineata sp. n.

Taxon classificationAnimaliaLepidopteraPyralidae

http://zoobank.org/31953FC2-6002-496D-8CE1-F24EA7E67D0C

Figs 4 , 9 , 14

#### Type material.

***Holotype***: ♂ – **CHINA**, Lao’an (30.33°N, 119.4°E), Mt. Tianmu, Zhejiang, 555 m, 5.VII.2014, coll. Aihui Yin, Xuemei Hu and Qingyun Wang, slide No. RH15430.

***Paratypes***: (17♂, 3♀) – **Guizhou**: 1♂, Neila, Limingguan, Libo County, 800 m, 18.VII.2015, coll. Meiqing Yang and Gaeun Lee, slide No. RH15425; 1♂, Limingguan, Libo County, 820 m, 21.VII.2015, coll. Meiqing Yang and Gaeun Lee, slide No. RH15428; **Hainan**: 1♀, Diaoluoshan Nature Reserves, 922 m, 26.V.2015, coll. Peixin Cong, Wei Guan and Sha Hu, slide No. RH16152; **Jiangxi**: 1♀, Mt. Jiulian, 21.VII.2006, coll. Jiasheng Xu and Weichun Li; **Zhejiang**: 2♂, Mt. Tianmu, 325 m, 26.VI.2013, coll. Aihui Yin and Xiuchun Wang; 5♂, Huangtanyu, Mt. Jiulong, 467 m, 3–9.VII.2013, coll. Aihui Yin and Xiuchun Wang; 1♂, Yanping, Mt. Jiulong, 530 m, 4.VII.2013, coll. Aihui Yin and Xiuchun Wang; 2♂, Zhangkengkou, Mt. Jiulong, 623 m, 5.VII.2013, coll. Aihui Yin and Xiuchun Wang; 3♂, Jiufu, Mt. Longtang, 520 m, 26.VII.2014, coll. Aihui Yin, Xuemei Hu and Qingyun Wang, slide No. RH15247;  1♂, Mt. Tianmu, 335 m, 20.VII.2015, coll. Aihui Yin, Kang Lou and Tao Wang, slide No. RH15429; 1♀, Laofoyan, Shuangxikou, Jiangshan, 424 m, 7.VIII.2016, coll. Qingyun Wang, Meiqing Yang and Ping Liu; 1♂, Linkeng, Yongjia, 387 m, 24.VIII.2016, coll. Qingyun Wang, Meiqing Yang and Ping Liu, slide No. RH16455.

#### Diagnosis.

This species is similar to *L. subtrapezia* sp. n. in both forewing pattern and male genitalia. It can be distinguished by the long scape extension apically reaching the mesothorax; in male genitalia by the straight posterior margin of the juxta and the very short cornutus approx 1/14 length of the phallus. In *L. subtrapezia* sp. n., the extremely short sub-globose scape extension doesn’t apically reach the patagium; the posterior margin of the juxta is concave medially and the long cornutus is approx 2/7 length of the phallus.

#### Description.

Adult (Fig. 4) wingspan 41.0–46.0 mm. Head earthy yellow in male, yellowish white in female. Male labial palpus white on inner side, grayish green mixed with reddish fuscous on outer side; first segment half length of second; second segment reaching vertex apically, inner side with dense long hair-like scales white on basal half and grayish green on distal half; third segment approx 1/6 length of second, tapering. Female labial palpus slightly porrect, widened distally, white on inner side, reddish fuscous mixed with black on outer side; first segment approx 1/4 length of second; second segment with inner side bearing long white hair-like scales on distal half; third segment approx 1/5 length of second, tapering. Maxillary palpus compressed, short, white mixed with reddish fuscous and grayish green. Antenna with ventral surface yellowish fuscous, with short pale gray cilia longer in male than in female; dorsal surface dirty white mixed with fuscous; scape extension strongly developed, reaching mesothorax, dirty white mixed with reddish fuscous, also with grayish green basally, with long dirty white mixed with reddish fuscous hair-like scales ventrally and apically. Thorax and tegula dirty white mixed with black. Forewing with basal area dirty white mottled reddish fuscous, grayish green with reddish fuscous on anterior 1/4, mixed with black along dorsum; median area blackish gray, grayish green on anterior 1/4; distal area grayish green mixed with black; antemedian line black, wavy, extending from basal 1/3 of costal margin slightly oblique outward to before middle of dorsum; postmedian line slightly darker than median area, ill defined, serrate, from 2/3 of costal margin directed obliquely inward to anterior 1/5 from costa, then outward to M_3_, inward to CuP, finally straight to 5/6 of dorsum and forming a right angle with dorsum, edged with grayish white along outer margin; glandular swelling yellowish fuscous; longitudinal black line from base to before antemedian line below lower margin of cell, interrupted with white and black tuft beyond middle; discocellular spot small, indistinct, represented by black mixed with reddish fuscous tuft; terminal line dirty white, with uniform black rectangular spots evenly spaced along its inner side, interrupted with dirty white on veins. Hindwing gray, darkening from base toward apex; postmedian line dark gray, edged with dirty white along outer margin, ill-defined, extending from M_1_ slightly arched outward to 1A. Cilia of both wings pale pink, interrupted with black on extension of veins. Legs white on inner side; on outer side, fore femur dirty white mixed with reddish fuscous and grayish green, tibia and tarsus grayish green mixed with black, mid femur grayish green mixed with black, tibia dirty white mixed with grayish green, with dense long pale pink hair-like scales, tarsus black mixed with grayish green, hind femur grayish green mixed with black and reddish fuscous, tibia pale pink mixed with black, with dense long pale pink hair-like scales, tarsus black mixed with grayish green, all tarsi white at apex of each tarsomere. Abdomen black, mixed with white and grayish green, 3rd to 6th segments white posteriorly on dorsal surface.

Male genitalia (Fig. 9). Uncus sub-rectangular, approximately 1.8 times longer than wide, slightly widened distally, distal 1/3 with dense short setae dorsally. Gnathos lateral arms slender, joined from 3/5; distal process hooked. Valva approximately twice as long as maximum width, with dense long hairs; costa straight, reaching before apex of valva; ventral margin obtuse, curved band from ventrobasal corner to base of juxta approximately 1.3 times length of ventral margin. Vinculum approximately twice as long as uncus. Juxta horizontally extended outward in triangle proximally, basal 3/5 separated, lateral arms straight and parallel from near proximal base; distal 2/5 merged sub-rectangularly, straight on posterior margin; posterolateral lobe almost membranous, semicircular, with short hairs; slender lateral arm extending from basal 2/3 at right angle not reaching vinculum. Phallus membranous in basal 2/3, weakly sclerotized in distal 1/3, approximately 1.3 times length of valva; dorsal sclerotized plate sub-rounded, much smaller than ventral plate; cornutus very short, approx 1/14 length of phallus.

Female genitalia (Fig. 14). Papillae anales shovel-shaped, narrowly rounded posteriorly, with dense fine hairs. Eighth segment sub-rectangular, with sparse long hairs posteriorly; tergite almost membranous anteriorly and posteriorly; sternite narrow, membranous medially. Apophyses anteriores approximately 1.2 times length of apophyses posteriores. Antrum annular. Ductus bursae with basal 3/5 straight, distal 2/5 S shaped, with longitudinal ridges. Corpus bursae sub-circular, approx 2/5 length of ductus bursae; signa long, ovate, with longitudinal ridge along central line.

#### Distribution.

China (Guizhou, Hainan, Jiangxi, Zhejiang).

#### Etymology.

The specific name is derived from the Latin *nigr*- and *lineatus*, referring to the forewing with black line from the base to before the antemedian line below the lower margin of the cell.

### Locastra subtrapezia sp. n.

Taxon classificationAnimaliaLepidopteraPyralidae

http://zoobank.org/3B4AFB80-B8A3-4331-8686-653E97BE3433

Figs 5 , 10

#### Type material.

***Holotype***: ♂ – **CHINA**, Huaping (25.6°N, 109.9°E), Guangxi, 950 m, 7.VIII.2006, coll. Weichun Li, slide No. WYP05141.

***Paratypes***: (3♂) – **Guizhou**: 1♂, Heiwan, Jiangkou, 600 m, 28.VII.2001, coll. Houhun Li and Xinpu Wang, slide No. WYP06033; 1♂, Maolan Nature Reserves, 1.IX.2011, coll. Jinwei Li, slide No. RH16457; **Zhejiang**: 1♂, Jiufu, Mt. Longtang, 520 m, 26.VII.2014, coll. Aihui Yin, Xuemei Hu and Qingyun Wang, slide No. RH15248.

#### Diagnosis.

This species is characterized by the juxta sub-trapezoidal in distal 1/3. It is similar to the previous species *L. nigrilineata* sp. n. in both forewing pattern and male genitalia. The differences between the two species are given under *L. nigrilineata* sp. n.

#### Description.

Adult (Fig. 5) wingspan 38.0–42.0 mm. Frons grayish green, vertex yellowish fuscous. Labial palpus grayish green mixed with white and black; first segment approx 1/3 length of second; second segment reaching vertex apically, inner side with hair-like scales grayish green on basal half, yellowish fuscous on distal half; third segment 1/3 length of second, tapering. Maxillary palpus compressed, short, dirty white on inner side, grayish green on outer side. Antenna with ventral surface yellowish fuscous, with short pale gray cilia; dorsal surface dirty white mixed with black; scape extension extremely short, sub-globose, reaching before patagium, grayish green. Thorax and tegula dirty white mixed with yellowish fuscous and black. Forewing grayish green mixed with black, slightly paler near termen; basal area with posterior half dirty white, suffused with reddish fuscous and black scales; antemedian line black, wavy, running from 2/5 of costal margin obliquely outward to before middle of dorsum; postmedian line black (worn), serrate, from 2/3 of costal margin oblique inward to anterior 1/5 from costa, then outward to M_3_, inward to CuP, finally straight to 3/4 of dorsum, edged with dirty white along its outer margin; glandular swelling yellowish white; discocellular spot small, represented by black mixed with fuscous tuft; black tuft placed below 2/3 of lower margin of cell, with white scales on inner edge; terminal line white, with uniform black mixed with reddish fuscous narrow rectangular spots evenly spaced along its inner side, interrupted with white on veins. Hindwing gray, darkening from base toward apex; postmedian line gray, extending from middle of M_1_ slightly arched outward to CuA_2_, edged with obscure grayish white along its outer margin. Cilia of both wings grayish white with pale red, interrupted with black on extension of veins. Legs grayish white mixed with fuscous and grayish green on inner side; on outer side, fore femur white mixed with grayish green, tibia and tarsus black mixed with grayish green, mid leg black mixed with grayish green, tibia with long black mixed with white hair-like scales, hind leg black mixed with grayish green, tibia with long grayish white hair-like scales, all tarsi white at apex of each tarsomere.

Male genitalia (Fig. 10). Uncus sub-rectangular, approximately 1.5 times as long as wide, slightly widened distally, distal 1/3 with dense short setae dorsally. Gnathos arms slender, joined from 2/3; distal process hooked. Valva approximately 1.6 times as long as maximum width, with dense long hairs; costa reaching before apex of valva; ventral margin obtusely arched, curved band from ventrobasal corner to base of juxta as long as ventral margin. Vinculum ap approximately approximately 2.0 times as long as uncus. Juxta with basal 2/3 separated, lateral arms widely apart basally, extending inward from base to basal 2/3, then outward before mergence; distal 1/3 merged sub-trapezoidally, concave medially on posterior margin; posterolateral lobe almost membranous, sub-triangular, with short hairs; slender lateral arm extending from basal 2/3 at right angle to vinculum. Phallus narrower medially, approximately 1.4 times length of valva; sclerotized dorsal plate sub-rounded, smaller than ventral plate; cornutus long, hooked, approx 2/7 length of phallus.

Female unknown.

#### Distribution.

China (Guangxi, Guizhou, Zhejiang).

#### Etymology.

The specific name is derived from the Latin *sub*- and *trapezius*, referring to the shape of the juxta in distal 1/3.

### Locastra viridis sp. n.

Taxon classificationAnimaliaLepidopteraPyralidae

http://zoobank.org/761B4764-3A87-45D3-AB5A-D166CEC36F51

Figs 6 , 11 , 15

#### Type material.

***Holotype***: ♂ – **CHINA**, Xiajinchang (23°10'N, 104°48'E), Malipo County, Wenshan, Yunnan, 1470 m, 26.VII.2016, coll. Kaijian Teng, Gaeun Lee and Tao Wang.

***Paratypes***: (24♂, 2♀) – **Guangxi**: 8♂, Yangmei’ao, Huajiang County, Hechi, 1180 m, 23–26.VII.2015, Meiqing Yang and Gaeun Lee, slide Nos. RH15427, RH15321; **Henan**: 3♂, Mt. Baiyun, Luoyang, 1560 m, 22.VII.2001, coll. Dandan Zhang, slide Nos. WYP05074, WYP06031; 1♂, 1♀, Mt. Wangwu, Jiyuan, 800 m, 30.VII.2006, coll. Hui Zhen and Denghui Kuang, slide No. WYP06036♀; **Hubei**: 1♂, Shayuan, Hefeng, 1260 m, 18.VII.1999, coll. Houhun Li *et al*., slide No. WYP06034; 1♂, Bapingying, Xianfeng, 1280 m, 22.VII.1999, coll. Houhun Li *et al*., slide No. WYP05077; **Yunnan**: 6♂, 1♀, 26–29.VII.2016, other data same as holotype, slide Nos. RH16459♂, RH16460♀; 4♂, Taiyanghe Nature Reserves, Pu’er, 1450 m, 14.VII.2016, coll. Kaijian Teng, Gaeun Lee and Tao Wang.

#### Diagnosis.

This species is similar to *L. muscosalis* in male genitalia. It can be distinguished by the sub-rectangular uncus, the valva without a process at the base of the ventral margin, and the juxta concave in V shape on the posterior margin; the sub-rectangular antrum and the corpus bursae approx 2/5 as long as the ductus bursae. In *L. muscosalis*, the uncus is inverted sub-trapezoidal, the valva bears a triangular process at basal 1/3 of the ventral margin (Fig. 13), the juxta concave in U shape on the posterior margin; the antrum is narrower, and the shorter corpus bursae is approx 1/3 as long as the ductus bursae.

#### Description.

Adult (Fig. 6) wingspan 29.0–38.0 mm. Head black mixed with grayish green. Male labial palpus pale grayish green, mixed with black on outer side; first segment half length of second; second segment reaching vertex, inner side with blackish green hair-like scales grayish white basally; third segment black, approx 1/4 length of second, tapering. Female labial palpus shorter than in male, slightly porrect; second segment with short grayish white hair-like scales on ventral surface; third segment approx 2/3 length of second. Maxillary palpus compressed, short, grayish green mixed with black. Antenna with ventral surface yellowish fuscous, with short pale gray cilia denser in male than in female; dorsal surface white mixed with black; scape extension extremely short, sub-globose, reaching before patagium, with grayish green scales. Thorax and tegula grayish green mixed with black and yellowish fuscous. Forewing with basal area black mixed with grayish green and fuscous; median area fuscous mixed with black, pale green mixed with black along costal margin; distal area pale green mixed with black; antemedian line black, wavy, running from 1/3 of costal margin slightly oblique outward to 2/5 of dorsum, edged with pale green fascia on both inner and outer margins; postmedian line black, serrate, running from 2/3 of costal margin oblique outward to CuA_1_, then inward to CuP, finally slightly outward to 4/5 of dorsum; glandular swelling small, yellowish fuscous, surrounded by black scales; black tuft placed below middle of lower margin of cell; discocellular spot small, represented by fuscous mixed with black tuft; terminal line grayish white, with uniform black rectangular spots evenly spaced along its inner side, interrupted with grayish white on veins. Hindwing gray, darkening from base toward apex; postmedian line ill-defined, blackish gray, serrate, extending from 2/3 of costal margin oblique outward to CuA_2_, edged with grayish white along its outer margin. Cilia of both wings pale grayish green, interrupted with black on extension of veins. Legs grayish white on inner side; on outer side black mixed with grayish green, fore tibia with grayish green hair-like scales, mid tibia with dense black mixed with grayish green hair-like scales, hind tibia with sparse grayish white mixed with grayish green hair-like scales, all tarsi white at apex of each tarsomere. Abdomen grayish white mixed with black on ventral surface; dorsal surface with 1st and 2nd segments yellowish fuscous, suffused with white and black scales, remaining segments black, suffused with white and yellowish fuscous scales.

Male genitalia (Fig. 11). Uncus sub-rectangular, app approximately rox 1.7 times as long as wide; distal 1/3 slightly widened, with dense short setae dorsally. Gnathos arms slender, joined from 3/5; distal process hooked, narrowed to pointed apex. Valva approximately 1.6 times as long as maximum width, with dense long hairs; costa reaching apex of valva; ventral margin arched obtusely, with narrow band from ventrobasal corner to base of juxta half length of ventral margin. Vinculum approximately as long as uncus. Juxta dilated proximally, its basal half separated, lateral arms relatively wide, sub-parallel; distal half merged sub-rectangularly, concav in U shape on posterior margin; posterolateral lobe membranous, elongate triangular, with short hairs; slender lateral arm extending from middle at 60° angle to vinculum. Phallus approximately 1.7 times as long as maximum length of valva; sclerotized sub-ovate dorsal plate smaller; cornutus long, slightly curved ventrad.

Female genitalia (Fig. 15). Papillae anales sub-triangular, obtuse posteriorly, with dense long hairs. Eighth tergite shallowly concave anterior margin medially; sternite sub-rectangular, membranous medially, with sparse setae on posterior margin. Apophyses anteriores slightly longer than apophyses posteriores, with basal 1/5 expanded triangularly; apophyses posteriores thinner, basal 1/5 slightly wavy. Antrum sub-rectangular, approx 1/2 as long as wide, weakly sclerotized. Ductus bursae membranous, basal 1/3 wide, distal 2/3 narrower, curved, with longitudinal ridges. Corpus bursae ovate, approx 2/5 length of ductus bursae; signa sub-ovate, with semicircular ridge medially, serrate on one edge.

#### Distribution.

China (Guangxi, Henan, Hubei, Yunnan).

#### Etymology.

The specific name is derived from the Latin *viridis*, referring to the forewing with more green scales.

### Locastra solivaga sp. n.

Taxon classificationAnimaliaLepidopteraPyralidae

http://zoobank.org/609975D1-CEAB-4D9A-8966-DBAEF8FAE4A4

Figs 7 , 12

#### Type material.

***Holotype***: ♂ – **CHINA**, Laofoyan (28°21'N, 118°41'E), Shuangxikou, Jiangshan, Zhejiang, 424 m, 7.VIII.2016, coll. Qingyun Wang, Meiqing Yang and Ping Liu, slide No. RH16458.

***Paratype***: – **Fujian**: 1♂, Guadun, Wuyishan, 1100 m, 29.VII.2008, coll. Weichun Li, Yongling Sun and Haiyan Bai, slide No. RH16484.

#### Diagnosis.

This species is similar to *L. muscosalis* in the forewing pattern. It can be distinguished in male genitalia by the sub-rectangular uncus nearly 5.0 times as long as wide at maximum and the valva without a process at the base of the ventral margin. In *L. muscosalis*, the inverted sub-trapezoidal uncus is approximately 1.5 times as long as wide at maximum, and the valva bears a triangular process at the base of the ventral margin (Fig. 13).

#### Description.

Adult (Fig. 7) wingspan 31.0–33.0 mm. Frons black, vertex yellowish fuscous mixed with yellowish white. Labial palpus grayish green mixed with black; first segment white basally, approx 1/4 length of second; second segment reaching vertex apically, inner side with dark grayish green hair-like scales shortening from base to tip; third segment short, approx 1/7 length of second, tapering, dirty white at apex. Maxillary palpus compressed, short, white mixed with fuscous on basal half, grayish green on distal half. Antenna with ventral surface brownish yellow, with short pale gray cilia; dorsal surface white; scape extension extremely short, sub-globose, reaching before patagium, with yellowish fuscous scales. Thorax and tegula dirty white mixed with black, or black mixed with dirty white and fuscous. Forewing with basal 1/3 black, mixed with reddish fuscous on posterior half; distal 2/3 pale grayish green, suffused with black and reddish fuscous scales, with more reddish fuscous scales near dorsum medially; antemedian line black, extending from 1/3 of costal margin slightly arched outward to near middle of wing, then arched outward to 2/5 of dorsum; postmedian line black, running from 2/3 of costal margin, slightly arched outward to anterior 1/4 from costa, then obliquely outward to CuA_1_, inward to CuP, finally straight to 3/4 of dorsum; glandular swelling large, yellowish fuscous; black mixed with reddish fuscous tuft placed below middle of lower margin of cell; discocellular spot small, represented by black mixed with reddish fuscous tuft; terminal line dirty white, with uniform black rectangular spots evenly spaced along its inner side, interrupted with dirty white on veins. Hindwing gray, darkening from base toward apex; postmedian line ill-defined, grayish white, running from 2/3 of costal margin obliquely outward to CuA_2_, then inward to 2A. Cilia of both wings grayish white with pale red, interrupted with black on extension of veins. Legs grayish white on inner side; on outer side, fore femur white mixed with black and reddish fuscous, tibia black mixed with grayish green and fuscous, tarsus grayish green mixed with black, mid femur black mixed with fuscous, tibia with basal 2/3 black, distal 1/3 white mixed with grayish green, with grayish white hair-like scales, tarsus grayish green mixed with black, hind leg black mixed with white, tibia with grayish white hair-like scales, all tarsi white at apex of each tarsomere.

Male genitalia (Fig. 12). Uncus narrowly elongate, nearly 5.0 times longer than wide, slightly narrowed medially, obtuse apically, distal 1/3 with dense short setae dorsally. Gnathos arms slender, joined from 3/5; distal process tapering to hooked apex. Valva relatively narrow, approximately 3.0 times as long as maximum width, with dense long hairs; costa reaching apex of valva; ventral margin obtusely arched, with narrow band from ventrobasal corner to base of juxta approximately 0.7 times length of ventral margin. Vinculum as long as uncus. Juxta dilated proximally, basal 2/5 separated, lateral arms straight and parallel; distal 3/5 merged sub-rectangularly, concave in V shape on posterior margin; posterolateral lobe sub-rectangular, almost membranous, with short hairs; slender lateral arm extending from middle at right angle to vinculum. Phallus approximately as long as valva; sub-ovate plates subapical, dorsal plate smaller; cornutus long, slightly curved, distally enlarged.

Female unknown.

#### Distribution.

China (Fujian, Zhejiang).

#### Etymology.

The specific name is derived from the Latin *solivagus*, referring to the narrow and elongate uncus.

### Locastra muscosalis

Taxon classificationAnimaliaLepidopteraPyralidae

(Walker, 1866)

Figs 8 , 13 , 16

Taurica muscosalis Walker, 1866: 1269. Type locality: North China.
Taurica sikkima Moore, 1888: 202.
Stericta sikkima Snellen, 1890: 563.
Locastra cristalis Hampson, 1893: 157.
Locastra muscosalis (Walker, 1866): Mutuura 1957: 105.

#### Material examined.

(241♂, 53♀) **. CHINA, Fujian**: 1♂, Chishuizhan, Mt. Daiyun, 1015 m, 22.V.2012, coll. Jinwei Li, slide No. RH16463; **Guangdong**: 1♂, Mt. Dadong, Lian County, 5.VII.2008, coll. Fengxia He (SYSBM); 1♂, Niupoling, Yangchun, 18.VIII.2009, coll. Fengxia He, slide No. RH16461; 1♂, Heishiding, Fengkai, 1.VII.2010, coll. Haidong Chen, Dandan Zhang and Bo Tong (SYSBM); 1♂, Guangming, Fengkai, 2.VII.2010, coll. Haidong Chen, Dandan Zhang and Bo Tong (SYSBM); 1♂, Guangming, Fengkai, 14.VIII.2010, coll. Haidong Chen, Dandan Zhang and Bo Tong (SYSBM); 1♂, Mt. Danxia, Shaoguan, 96 m, 7.VI.2012, coll. Jinwei Li (SYSBM); **Guangxi**: 1♂, Jiman, Anchui, Rongshui, 650 m, 16.VII.2004, coll. Jiasheng Xu; 24♂, Huaping, 950 m, 1–8.VIII.2006, coll. Weichun Li, slide No. WYP06032; 13♂, Mt. Yuanbao, 700 m, 10–12.VIII.2006, coll. Weichun Li; 2♂, Longrui Nature Reserves, 18–19.VIII.2011, coll. Dandan Zhang (SYSBM); 1♂, Longrui Nature Reserves, 19.VIII.2011, coll. Muchun Cheng (SYSBM); 1♂, Nonggang Nature Reserves, 20.VIII.2011, coll. Dandan Zhang (SYSBM); 1♂, Nonggang Nature Reserves, 20.VIII.2011, coll. Muchun Cheng (SYSBM); 1♀, Peixiu, Rongshui, 30.VIII.2011, coll. Jinwei Li (SYSBM); 1♂, Mt. Dayao, Jinxiu, 561 m, 7.VII.2013, coll. Xiaohua Chen (SYSBM); 9♂, 1♀, Hekou, Mt. Dayao, Jinxiu, 823 m, 18–20.VII.2015, coll. Mujie Qi and Shengnan Zhao, slide Nos. RH15423♂, RH15424♀; 1♂, Jiuniutang, Mt. Mao’er, Guilin, 1012 m, 23.VII.2015, coll. Mujie Qi and Shengnan Zhao; 1♂, Yangmei’ao, Huanjian County, Hechi, 1180 m, 23.VII.2015, coll. Meiqing Yang and Gaeun Lee; 2♂, Yangmei’ao, Huanjian County, Hechi, 1180 m, 24.VII.2015, coll. Meiqing Yang and Gaeun Lee; **Guizhou**: 8♂, Daheba, Mayanghe, 430 m, 10.VI.2007, coll. Xicui Du; 2♂, 2♀, Mt. Fanjing, Jiangkou County, 26.VIII.2012, coll. Jinwei Li and Xiaohua Chen (SYSBM); 1♂, Taojiang, Leishan County, 27.VIII.2012, coll. Jinwei Li and Xiaohua Chen (SYSBM); 5♂, 1♀, Maolan Nature Reserves, 797 m, 12.VII.2013, coll. Xiaohua Chen (SYSBM); 1♂, Mt. Leigong, 1198 m, 14.VII.2013, coll. Xiaohua Chen (SYSBM); 1♂, Neila, Limingguan, Libo County, 800 m, 18.VII.2015, coll. Meiqing Yang and Gaeun Lee, slide No. RH15426; 11♂, 2♀, Dongdai, Limingguan, Libo County, 720 m, 19.VII 2015, coll. Meiqing Yang and Gaeun Lee; 15♂, Pobao, Limingguan, Libo County, 740 m, 20.VII.2015, coll. Meiqing Yang and Gaeun Lee; **Hainan**: 1♂, Jianfengling Nature Reserves, 143 m, 6.IX.2013, coll. Weicai Xie (SYSBM); 2♂, Luoshuai, Yuanmen, Baisha County, 284 m, 18.V.2013, coll. Jinwei Li (SYSBM); 2♂, Hongxin, Yuanmen, Baisha County, 460 m, 1.VII.2014, coll. Peixin Cong, Linjie Liu and Sha Hu; 1♂, Wuzhishan Nature Reserves, 742 m, 5.VII.2014, coll. Peixin Cong, Linjie Liu and Sha Hu; 6♂, 1♀, Sanfenzhan, Mt. Limu, 240 m, 26–27.VII.2014, coll. Peixin Cong, Linjie Liu and Sha Hu, slide No. RH15249♂; 3♂, Wuzhishan Nature Reserves, 742 m, 19.V.2015, coll. Peixin Cong, Linjie Liu and Sha Hu; 2♂, Diaoluoshan Nature Reserves, 922 m, 24–25.V.2015, coll. Peixin Cong, Linjie Liu and Sha Hu; 2♂, Hongkan, Yinggeling, 508 m, 15–17.VI.2015, coll. Peixin Cong, Wei Guan and Sha Hu; 1♂, Wuzhishan Nature Reserves, 738 m, 4.VII.2015, coll. Qingyun Wang, Suran Li and Mengting Chen; 1♂, Yajia, Bawangling, 261 m, 20.VII.2015, coll. Qingyun Wang, Suran Li and Mengting Chen; 1♂, Hongxin, Yuanmen, Baisha County, 445 m, 31.VII.2015, coll. Qingyun Wang, Suran Li and Mengting Chen; 3♂, 3♀, Limushan Forest Park, 607 m, 20.VII.2016, coll. Xia Bai, Shuonan Qian and Wanding Qi, slide No. RH16454♀; 15♂, 1♀, Wuzhishan Nature Reserves, 738 m, 22–30.VII.2016, coll. Xia Bai, Shuonan Qian and Wanding Qi; 3♂, Lizu Hall, Shuiman, Wuzhishan, 766 m, 2.VIII.2016, coll. Xia Bai, Shuonan Qian and Wanding Qi; 4♂, 1♀, Nankai, Yinggeling, 210 m, 11–14.VIII.2016, coll. Xia Bai, Shuonan Qian and Wanding Qi; 2♂, Tianchi, Jianfengling, 787 m, 9–10.VIII.2016, coll. Xia Bai, Shuonan Qian and Wanding Qi; **Hebei**: 4♂, 1♀, Shuangyuanfeng, Mt. Wuling, Xinglong County, 800 m, 15–29.VII.2011, coll. Houhun Li and Yanpeng Cai; **Henan**: 10♂, 1♀, Mt. Jigong, Xinyang, 700 m, 13–15.VII.2001, coll. Dandan Zhang, slide Nos. WYP05047♂, WYP05140♀; 1♂, Shuiliandong, Tongbai, 300 m, 16.VII.2001, coll. Dandan Zhang; 1♂, Shiziping, Lushi, 1200 m, 19.VII.2001, coll. Dandan Zhang, slide No. WYP05048; 1♂, Shibanyan, Linzhou, 550 m, 22.VII.2006, coll. Hui Zhen and Denghui Kuang; 2♂, Xiuwu, Mt. Yuntai, Jiaozuo, 1028 m, 7–9.VIII.2014, coll. Peixin Cong, Linjie Liu and Sha Hu, slide No. RH15245; **Hubei**: 2♂, Maoba, Lichuan, 700 m, 30.VII.1999, coll. Houhun Li *et. al*.; **Hunan**: 5♂, 1♀, Yueyan, Dao County, 21–22.VIII.2012, coll. Jinwei Li and Xiaohua Chen, slide No. RH16462♂; 1♀, Zhupo, Huitong County, 23.VIII.2012, coll. Jinwei Li and Xiaohua Chen (SYSBM); **Jiangxi**: 1♂, 7♀, Mt. Jinpen, 18–19.VII.2006, coll. Jiasheng Xu and Weichun Li; 1♂, Xiangshan, 23.VII.2006, coll. Jiasheng Xu and Weichun Li, slide No. WYP06037; 3♀, Mt. Feng, 26.VII.2006, coll. Jiasheng Xu and Weichun Li;1♀, Panlong, Ganzhou, 28.VII.2006, coll. Jiasheng Xu and Weichun Li; **Liaoning**: 1♂, Laotuding, Huanren County, 30.VII.2012, coll. Dandan Zhang and Lijun Yang; 1♂, Mt. Bailang, Jianchang County, 658 m, 9.VIII.2016, coll. Mujie Qi, Juan Li and Yanyan Jia; **Shaanxi**: 1♂, Haopingsi, Yingtou, 1251 m, 17.VII.2012, coll. Jinwei Li (SYSBM); **Sichuan**: 1♂, Caoping, Sanjiang, Wenchuan, 1557 m, 9.VII.2014, coll. Kaijian Teng, Wei Guan, Xiuchun Wang and Shurong Liu; 2♂, Wanniansi, Mt. E’mei, 830 m, 13.VII.2014, coll. Kaijian Teng, Wei Guan, Xiuchun Wang and Shurong Liu; 1♂, Bifengxia, Ya’an, 1115 m, 28.VI.2016, coll. Kaijian Teng and Xiaofei Yang; 1♀, Hailuogou, Luding, 1695 m, 1.VII.2016, coll. Kaijian Teng and Xiaofei Yang, slide No. RH16464; **Tianjin**: 1♀, Mt. Jiulong, Ji County, 10–12.VII.2009, coll. Weichun Li; **Tibet**: 7♂, Motuo County, 1103 m, 8.VII.2013, coll. Jinwei Li (SYSBM); 1♂, 1♀, Dexing, Motuo County, 835 m, 9.VII.2013, coll. Jinwei Li (SYSBM); **Yunnan**: 1♂, Ruili Rare Botanic Garden, 1000 m, 7.VIII.2005, coll. Yingdang Ren, slide No. WYP06038; 1♂, Bubang, Mengla, 650 m, 22.VIII.2005, coll. Yingdang Ren, slide No. WYP06035; 2♂, Bawan, Baoshan, 1040 m, 9.VIII.2007, coll. Dandan Zhang (SYSBM); 1♂, Baihualing, Baoshan, 1520 m, 13.VIII.2007, coll. Dandan Zhang (SYSBM); 1♂, Baihualing, Baoshan, 1520 m, 13.VIII.2007, coll. Dayong Xue (SYSBM); 1♂, 1♀, Mt. Jizu, Binchuan, 1831 m, 29.VI.2012, coll. Jinwei Li (SYSBM); 1♀, Mt. Weibao, Dali, 2205 m, 1.VII.2014, coll. Kaijian Teng, Wei Guan, Xiuchun Wang and Shurong Liu, slide No. RH15246; 7♂, Mt. Jizu, Dali, 2228 m, 27–28.VII.2014, coll. Kaijian Teng, Wei Guan, Xiuchun Wang and Shurong Liu; 3♂, Baihualing, Baoshan, 1474 m, 5–7.VIII.2014, coll. Kaijian Teng, Shurong Liu and Hua Rong; 10♂, 1♀, Xiajinchang, Malipo County, Wenshan, 1470 m, 26–29.VII.2016, coll. Kaijian Teng, Gaeun Lee and Tao Wang; 2♂, Daluo, Menghai, Jinghong, 640 m, 2.VIII.2016, coll. Kaijian Teng, Gaeun Lee and Tao Wang; 1♂, Mt. Bulang, Menghai, Jinghong, 1178 m, 4.VIII.2016, coll. Kaijian Teng, Gaeun Lee and Tao Wang, slide No. RH16483; 1♂, Lvshilin, Menglun, Jinghong, 580 m, 6.VIII.2016, coll. Kaijian Teng, Gaeun Lee and Tao Wang; **Zhejiang**: 1♀, Mt. Tianmu, 325 m, 28.VI.2013, coll. Aihui Yin and Xiuchun Wang; 1♂, Zhangkengkou, Mt. Jiulong, 623 m, 5.VII.2013, coll. Aihui Yin and Xiuchun Wang; 2♂, Huangtanyu, Mt. Jiulong, 467 m, 6.VII.2013, coll. Aihui Yin and Xiuchun Wang; 3♂, Lao’an, Mt. Tianmu, 555 m, 3.VII.2014, coll. Aihun Yin, Xuemei Hu and Qingyun Wang; 1♂, Mt. Tianmu, 335 m, 19.VII.2015, coll. Aihun Yin, Kang Lou and Tao Wang, slide No. RH15322; 1♀, Simingshan National Forest Park, Ningbo, 822 m, 31.VII.2016, coll. Qingyun Wang, Meiqing Yang and Ping Liu; 3♂, 17♀, Taohuadao, Zhoushan, 62.9 m, 4.VIII.2016, coll. Qingyun Wang, Meiqing Yang and Ping Liu; 1♀, Huangtianhu, Jingning, 787 m, 11.VIII.2016, coll. Qingyun Wang, Meiqing Yang and Ping Liu, slide No. RH16456; 1♀, Baishanzu Nature Reserves, Qingyuan, 1149 m, 14.VIII.2016, coll. Qingyun Wang, Meiqing Yang and Ping Liu.

#### Diagnosis.

Adult (Fig. 8) wingspan 32.0–41.0 mm. This species is characterized by the uncus inverted trapezoidal and the valva with a triangular process at basal 1/3 of the ventral margin (Fig. 13). It is similar to *L. solivaga* sp. n. in the forewing pattern and to *L. viridis* sp. n. in the male genitalia. The differences between the three species are stated above under the treatments of the latter two species.

#### Variation.

Specimens collected from Guizhou and Guangxi are darker than those from other localities.

#### Host plants.

Anacardiaceae: *Pistacia chinensis* Bunge, *Rhus chinensis* Mill., *R. sylvestris* Sieb. *et* Zucc., *R. verniciflua* Stokes; Juglandaceae: *Juglans mandshurica* Maxim., J. mandshurica var. sieboldiana Makino, *J. sinensis* (C. DC.) Dode., *Pterocarya stenoptera* C. DC.; Simaroubaceae: *Ailanthus altissima* (Mill.) Swingle (Bae and Paek 2006).

#### Distribution.

China (Fujian, Guangdong, Guangxi, Hainan, Hebei, Henan, Hongkong, Hubei, Hunan, Jiangxi, Liaoning, Shaanxi, Sichuan, Tianjin, Tibet, Yunnan, Zhejiang), Japan, India, Sri Lanka (Wang et al. 2003).

#### Remarks.

Walker (1866) described this species based upon the external characters, and our specimens match the wing pattern of his description. *Locastra muscosalis* resembles *L. crassipennis* in the male genitalia, as given by Janse (1931), but can be distinguished from the latter by its short and sub-globose scape extension distinct from that of *L. crassipennis* which is large, very broad at the base, and sickle-shaped in the distal 2/3.

## Supplementary Material

XML Treatment for Locastra

XML Treatment for Locastra nigrilineata

XML Treatment for Locastra subtrapezia

XML Treatment for Locastra viridis

XML Treatment for Locastra solivaga

XML Treatment for Locastra muscosalis
